# Development of Novel Antibacterial Ti-Nb-Ga Alloys with Low Stiffness for Medical Implant Applications

**DOI:** 10.3390/jfb15060167

**Published:** 2024-06-17

**Authors:** Rhianna McHendrie, Ngoc Huu Nguyen, Manh Tuong Nguyen, Khosro Fallahnezhad, Krasimir Vasilev, Vi Khanh Truong, Reza Hashemi

**Affiliations:** 1 College of Science and Engineering, Flinders University, Tonsley, SA 5042, Australia; 2 College of Medicine and Public Health, Flinders University, Bedford Park, SA 5042, Australia

**Keywords:** titanium alloys, gallium, antibacterial biomaterials, medical implants

## Abstract

With the rising demand for medical implants and the dominance of implant-associated failures including infections, extensive research has been prompted into the development of novel biomaterials that can offer desirable characteristics. This study develops and evaluates new titanium-based alloys containing gallium additions with the aim of offering beneficial antibacterial properties while having a reduced stiffness level to minimise the effect of stress shielding when in contact with bone. The focus is on the microstructure, mechanical properties, antimicrobial activity, and cytocompatibility to inform the suitability of the designed alloys as biometals. Novel Ti-33Nb-xGa alloys (x = 3, 5 wt%) were produced via casting followed by homogenisation treatment, where all results were compared to the currently employed alloy Ti-6Al-4V. Optical microscopy, scanning electron microscopy (SEM), and energy dispersive spectroscopy (EDS) results depicted a single beta (β) phase microstructure in both Ga-containing alloys, where Ti-33Nb-5Ga was also dominated by dendritic alpha (α) phase grains in a β-phase matrix. EDS analysis indicated that the α-phase dendrites in Ti-33Nb-5Ga were enriched with titanium, while the β-phase was richer in niobium and gallium elements. Mechanical properties were measured using nanoindentation and microhardness methods, where the Young’s modulus for Ti-33Nb-3Ga and Ti-33Nb-5Ga was found to be 75.4 ± 2.4 and 67.2 ± 1.6 GPa, respectively, a significant reduction of 37% and 44% with respect to Ti-6Al-4V. This reduction helps address the disproportionate Young’s modulus between titanium implant components and cortical bone. Importantly, both alloys successfully achieved superior antimicrobial properties against Gram-negative *P. aeruginosa* and Gram-positive *S. aureus* bacteria. Antibacterial efficacy was noted at up to 90 ± 5% for the 3 wt% alloy and 95 ± 3% for the 5 wt% alloy. These findings signify a substantial enhancement of the antimicrobial performance when compared to Ti-6Al-4V which exhibited very small rates (up to 6.3 ± 1.5%). No cytotoxicity was observed in hGF cell lines over 24 h. Cell morphology and cytoskeleton distribution appeared to depict typical morphology with a prominent nucleus, elongated fibroblastic spindle-shaped morphology, and F-actin filamentous stress fibres in a well-defined structure of parallel bundles along the cellular axis. The developed alloys in this work have shown very promising results and are suggested to be further examined towards the use of orthopaedic implant components.

## 1. Introduction

The increasing utilisation of implantable medical devices such as orthopaedic implants, attributed to an aging population and the heightened prevalence of related health conditions, has resulted in a greater occurrence of device-associated infections [1]. The formation of biofilms on the surfaces of implants often culminates in chronic infections and subsequently implant failure [1]. Having garnered significant attention as biomedical materials, titanium (Ti) and its alloys depict advantageous properties, including a nominal Young’s modulus, high strength, excellent biocompatibility, and corrosion resistance [2,3,4]. These properties render titanium alloys particularly suitable for orthopaedic implants, such as total hip replacement systems [5,6,7]. Among titanium alloys, the α/β alloy Ti-6Al-4V is the clinical standard for orthopaedic implant applications. However, the substantial disparity between the Young’s modulus of Ti-6Al-4V (approximately 110 GPa) and that of cortical bone (5–30 GPa) can lead to stress shielding, which may result in implant failure and bone resorption [8,9]. Moreover, it is well recognised within literature that the release of significant quantities of aluminium (Al) and vanadium (V) ions from Ti-6Al-4V can cause severe cytotoxic effects, including neurodivergent diseases and genetic damage [10,11,12]. These issues have prompted the investigation of Ti-based alloys with a Young’s modulus closer to that of bone, along with the inclusion of biocompatible alloying elements for biomedical implant applications.

Recent advancements in titanium alloy compositions have significantly improved their mechanical properties, addressing the limitations associated with stress shielding. The development of new-generation β-type Ti alloys with body-centered cubic (BCC) crystal structures has resulted in reduced stiffness levels (Young’s moduli), making them more advantageous for implants in contact with bone. Such improvements are increasingly reported within the literature [3,8,9,13,14]. Ning et al. [15], for example, developed Ti-35Nb-5Zr with a stiffness of 58.8 GPa and successful cell proliferation. Tan et al. [16] investigated Ti-xNb-7Zr (x = 23, 28, 33 wt%) alloys, concluding that increasing Nb content stabilizes the β-phase microstructure, reducing the Young’s modulus to as little as 29 GPa. These findings underscore the advantages of increasing Nb content, where Hanada et al. [17] further clarified that Ti-Nb alloys achieve a minimum modulus of 65 GPa when Nb additions are approximately 40 wt%. Moreover, in physicochemical solutions, Ti-Nb based alloys commonly exhibit superior corrosion resistance, attributed to the formation of a defect-free passive layer like Nb_2_O_5_, contrasting with the defective passive layer of V_2_O_5_ present in Ti-6Al-4V alloys [18,19]. Despite such improvements, β-type Ti-Nb alloys still significantly differ in modulus from bone, which may instigate implant failure. Moreover, bacterial infections post-implantation remain a challenge in biomedical applications, urging the need for biofilm prevention to mitigate morbidity and mortality rates, which can dramatically increase following revision surgeries [20].

Recent research has highlighted gallium’s effective antimicrobial properties without inducing cytotoxic effects [13,21,22]. Although many of the studies investigated the effect of Ga-doped materials such as glass and nanoparticle coatings rather than Ti-based systems, the antibacterial activity has nonetheless been documented [22]. For instance, Valappil et al. [23] reported that Ga-doped phosphate-based glass exhibited potent antibacterial effects against five gram-negative and gram-positive bacteria strains, where even 1 mol% addition of Ga proved highly effective. Similarly, Cochis et al. [21] found that Ga-doped Ti inhibited *A. baumannii* more effectively than Ag-doped Ti, in addition to maintaining no cytotoxic effect. Additionally, Cochis et al. [13] later reported that even 1–2 wt% Ga into Ti-Al-Zr-Si alloys demonstrated robust antibacterial efficacy without inducing cytotoxic effects. While the alloying of gallium to Ti-Nb alloys has been examined concerning transformation temperature properties, its impact on the mechanical properties was not reported until Alberta et al. [8,24,25]. In investigating Ti-45Nb-xGa (x = 2, 4, 6, 8 wt%) alloys, it was revealed that the addition of 4 wt% Ga resulted in the most favourable mechanical properties, including a near 40% increase in strength compared to Ti-45Nb. An important consideration is whether titanium alloys with gallium additions could exhibit beneficial antibacterial properties. This highlights the necessity to investigate the impact of gallium additions on Ti-Nb alloys for their antibacterial efficacy and cytocompatibility.

This research therefore aims to design and develop novel β-type Ti-Nb alloys by incorporating gallium, with a focus on enhancing mechanical, antibacterial, and cytotoxicity properties. Specifically, the objectives are to elucidate the impact of gallium additions on the microstructure, mechanical, and antibacterial properties of the Ti-Nb alloy. The study aims to achieve antimicrobial efficacy without inducing cytotoxic effects. Additionally, the research seeks to attain a stiffness level in the developed alloys that is lower than that of the reference alloy (Ti-6Al-4V), thereby minimizing stress shielding effects and improving compatibility with bone tissue. By addressing these objectives, the research aims to evaluate the potential of the developed alloys as biomaterials for orthopaedic implants.

## 2. Materials and Methods

### 2.1. Alloy Composition Design

The compositions of novel titanium alloys were designed based on extensive evaluation of β-phase Ti-Nb alloys reported within the literature. New-generation Ti alloys have received widespread interest, where the Ti-Nb system and its alloys show among the most promising properties. This includes desired phase morphology, reduced Young’s modulus, corrosion resistance, and excellent biocompatibilities [2,8,14,16,24,25,26,27,28]. This underpins the reasoning for its incorporation into the master alloy in this study. The content of niobium within the master alloy was intentionally selected as 33 wt%, as Nb content above 30 wt% is likely to promote a single bcc β-phase [29]. This microstructure is known to improve mechanical properties, including to reduce the Young’s modulus [25]. The minimum modulus for binary Ti-Nb alloys is 65 GPa when Nb alloying additions are approximately 40 wt%; however, greater α’ phase was exhibited, which can be deleterious to the Young’s modulus [17]. This indicates that niobium content should probably be kept within the range of 30 to 40 wt% to exhibit optimised mechanical properties. Furthermore, by increasing the elemental niobium content, the Young’s modulus of Ti-xNb-7Zr (x = 23, 28, 33 wt%) alloys were further reduced in the 33 wt% Nb alloy at 29.0 GPa [16]. Through consideration of all rationales, the niobium content was therefore selected as 33 wt% within the master alloy.

The incorporation of minor alloying additions of gallium to the Ti-33Nb master alloy, at concentrations of 3 and 5 wt%, represent novelty in the literature. Compositions were systematically chosen based on the antibacterial effect of Ga seen for 1–2 wt% additions [13], and because Ga is an α-phase stabiliser and should therefore be kept low when alloyed. Between 2, 4, 6, and 8 wt% incorporations of gallium, the 4 wt% alloy showed the best combination of mechanical properties [25]. Based on these reasonings, alloying additions of 3 and 5 wt% Ga to the Ti-33Nb master alloy were designed, therefore affording Ti-33Nb-3Ga and Ti-33Nb-5Ga (wt%).

### 2.2. Production of Alloys and Sample Preparation

Alloys of composition Ti-33Nb-xGa (x = 3, 5 wt%, chemical composition provided in Table 1) were produced as cylindrical rods (diameter = 20 mm and thickness = 40 mm) by American Elements (Los Angeles, CA, USA) with high purity Ti, Nb, and Ga pieces. The alloys were subsequently cast as cylindrical rods and subject to post treatment processes. In the present study, the developed alloys were compared to the commonly used Ti-6Al-4V alloy, produced by Dynamet (Wyomissing, PA, USA), of chemical composition specified in Table 2. Alloy rods were sectioned using an ISOMET 1000 Precision Saw equipped with a diamond blade into 20 mm by 8 mm thickness disks (1× replicate per sample). The samples were subsequently mounted in cold-hardening resin followed by curing for 10 min. Mounted samples were mechanically ground using a Tegramin 25 polishing machine (Struers, Ballerup, Denmark) with resin bonded diamonds in a rigid disc (MD-Meso). Plane grinding was followed by the single fine grinding step, using MD-Largo paired with abrasive 9 μm diamond suspension. Subsequently, chemical-mechanical polishing was conducted using a mixture of colloidal silica (OP-S) and hydrogen peroxide (10–30%) with MD-Chem polishing disks. The reaction product generated between hydrogen peroxide and titanium is continuously removed from the surface of the alloy samples by the silica suspension, resulting in a surface free of mechanical deformation. Finally, samples were cleaned in an ultrasonic bath (5 min) and dried with condensed air. The fabricated alloys were referenced to Ti-6Al-4V, which was subject to the same sample preparation methods.

### 2.3. Microstructural Evaluation (Optical Microscopy, SEM/EDS)

Microstructures of the polished samples were observed using a Zeiss Axio Imager Z2 optical microscope (Zeiss, Oberkochen, Germany) under illumination with white light. Samples for optical microscopy were sectioned from the as-cast alloys and polished to a mirror-like finish, as described in Section 3.2.2. Objective lenses ranging from 5 to 50× magnification were used for imaging each sample, where a Zeiss Axiocam 305 colour camera was used to record the microstructures. Camera acquisition settings were adjusted prior to image collection.

The microstructures of the developed alloys were analysed by means of a scanning electron microscope (Inspect, Model: F50, FEI, Hillsboro, OR, USA) equipped with a secondary electron detector, concentric backscatter electron detector, and an EDAX energy dispersive spectroscopy (EDS) detector (Ametek, Philadelphia, PA, USA). Samples for SEM analysis were cut from the as-cast alloys, mechanically ground, and polished, as described in Section 3.2.2. SEM images were scanned at a working distance of 10 mm, an emission current of 178.3 μA, and an accelerating voltage of 20 kV.

### 2.4. Mechanical Testing

#### 2.4.1. Microhardness

Microhardness testing was conducted on polished, unembedded samples using a DuraScan-20 Vickers microhardness tester (Struers, Ballerup, Denmark) paired with a diamond pyramid indenter. Each sample was subject to seven measurements within a matrix at an established load of 5 HV (49.04 N) and a dwell time of 3 s.

#### 2.4.2. Nanoindentation

Given the size limitations of the as-cast samples, nanoindentation testing was performed to elucidate the mechanical properties of the alloys, as typical tensile testing requires large, dog-bone alloy morphologies. Nanoindentation testing was performed using an IBIS Nanoindentation System Model B (Fischer-Cripps Laboratories, Sydney, NSW, Australia) coupled with a Berkovich diamond indenter to determine the Young’s modulus of the alloys. The polished samples each received 16 measurements in a rectangular map (4 × 4), where the indentations were distanced by 15 μm to prevent the influence of residual strain between adjacent indentations. The applied load was kept constant at 100 mN, with a depth of 19.2 μm, and a working distance of approximately 65 μm. Measurements were taken at a contact force 10% of the maximum at an unloading rate of 2.5 mN/second.

### 2.5. Biological Characterisation

#### 2.5.1. Antimicrobial Activity Assay

*Staphylococcus aureus* ATCC 25,923 (*S. aureus*) and *Pseudomonas aeruginosa* ATCC 15,692 (*P. aeruginosa*) were obtained from glycerol stocks maintained at −80 °C. A loop-full of the stock bacteria was thereafter placed onto a Tryptic Soy Broth (TSB) agar plate and incubated overnight. On a subsequent day, a single colony was placed into a TSB medium for further incubation overnight. The cell density was measured using a spectrophotometer at a wavelength of 600 nm.

The samples were sectioned into 10 mm diameter and 3 mm thickness profiles using a diamond saw and subjected to a 5-min ultrasonic cleaning process. The specimens were labelled accordingly and carefully placed in a 24-well plate. Each well of the plate contained 500 µL of bacterial suspension with a concentration of 10^6^ CFU/mL in TSB, where specimens were positioned such that they were fully submerged in solution. Specimens were incubated at 37 °C for 6 and 18 h in a humidified chamber. Subsequently, the samples underwent three rinses with phosphate-buffered saline (PBS) and were transferred to a fresh 24-well plate. In this experiment, 1 mL of BacLight Live/Dead stain (Invitrogen, ThermoFisher, Boston, MA, USA) was added to each well. The stain comprises an equal mixture of Syto9 and Propidium Iodide (PI), resulting in a final concentration of 1.5 µL mL^−1^ in PBS. After a 15-min period of incubation in a dark environment at room temperature, fast imaging was performed using a Zeiss LSM880 confocal laser scanning microscope (Oberkochen, Germany). The emission and excitation spectra for PI and Syto9 were configured at wavelengths of 490/635 nm and 480/500 nm, respectively. The viable-to-non-viable cell ratio was quantified using ImageJ Fiji software (version 1.53a). Antibacterial efficacy was calculated from the formula in Equation (1).

(1) $$\mathrm{Antibacterial}\;\mathrm{percentage}=\frac{\mathrm{Dead}\;\mathrm{bacteria}}{\mathrm{Total}\;\mathrm{bacteria}}\;\times \;100\%\;$$

#### 2.5.2. Cytotoxicity Assay

The in-vitro cytocompatibility evaluation of Ti-33Nb-3Ga, Ti-33Nb-5Ga, and control Ti-6Al-4V alloys was performed using direct methods on the human gingival fibroblast cell line (hGF). The Human Gingival Fibroblast cell line was provided by Associate Professor Peter Zilm, University of Adelaide, Adelaide, SA, Australia. The sectioned pieces (10 × 3 mm, 3× replicates per sample) were sterilised within 80% ethanol for 15 min and rinsed three times with PBS. Cells were cultured in Dulbecco’s modified Eagles medium (DMEM/F-12 GlutaMAX supplement; ThermoFisher, Boston, MA, USA), 10% fetal bovine solution (FBS), and 1% of penicillin-streptomycin (ThermoFisher, Boston, MA, USA), which will hereby be referred to as the medium. For the cytotoxicity assay, hGF was washed with PBS and detached with trypsin (0.25%) in a 37 °C humidified incubator with 5% CO_2_ for 2 min. To neutralise the trypsin, medium was added (3 mL), collected into a sterilised tube (15 mL), and centrifuged (1000 rpm, 2 min), where the supernatant was subsequently removed, and the pellets resuspended in fresh medium (2 mL). From this suspension, 10 μL was mixed with 10 μL of trypan blue solution (ThermoFisher, Boston, MA, USA) and was evaluated using an automated cell counter (Invitrogen Countless 3 Automated Cell Counter). The material discs were placed in a 24 well plate and seeded with hGF at a density of 40,000 cells/mL. After 24 h of direct cell contact in a 37 °C humidified incubator with 5% CO_2_, 10 vol% MTT solution (3-(4,5-dimethylthiazol-2-yl)-2,5-diphenyl tetrazolium bromide) was introduced and incubated for 2 h. The supernatants were then discarded, DMSO added, and shaken for 15 min to dissolve the purple formazan. The absorbance of the samples was measured in a 96 well plate at a wavelength of 570 nm using a BioTek Synergy HTX Multimode Reader.

*F-actin Staining:* Cells were grown in a 24-well plate (40,000 cells/well) with material and subsequently incubated for 24 h in a 37 °C humidified incubator with 5% CO_2_. Samples were washed twice with PBS, and materials were fixed with paraformaldehyde solution (3.7%) for 15 min, followed by additional PBS washing. Triton X-100 (0.1%) in PBS was introduced to the fixed cells for 3–5 min to increase permeability and subsequently washed with PBS two times. A phalloidin-conjugate working solution was prepared and added, followed by incubation at room temperature for 40 min, and additional PBS washing. The mounting of specimens with DAPI (aqueous, fluoroshield ab104139) was conducted, where specimens were imaged using an Olympus IX83 inverted microscope.

#### 2.5.3. Statistical Analysis

Antibacterial activity and cytotoxicity assay results are expressed as means ± standard deviations. Statistical analyses were performed using Student’s *t*-test. Differences were considered statistically significant at *p* < 0.05.

## 3. Results

### 3.1. Microstructural Characterisation

**Figure 1:**
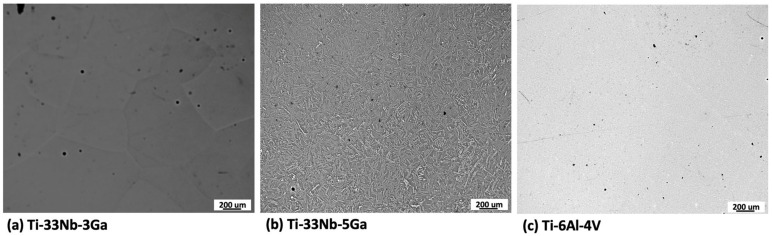
Optical micrographs of: (**a**) Ti-33Nb-3Ga, (**b**) Ti-33Nb-5Ga, and (**c**) Ti-6Al-4V.

The microstructures obtained for polished Ti-33Nb-3Ga, Ti-33Nb-5Ga, and Ti-6Al-4V alloys by optical microscopy are depicted in Figure 1a–c. Figure 1a shows the optical micrographs of the 3 wt% Ga alloy, which consists of equiaxed β-grains in varying grain sizes ranging from 300 to 800 μm. Comparatively, the microstructure of Ti-33Nb-5Ga in Figure 1b depicts α-phase dendritic branches encapsulated in β-phase equiaxed grains and grain boundaries, with the average grain size ranging from 100 to 400 μm. Furthermore, the microstructure of the Ti-6Al-4V reference alloy depicts homogenous grain distribution. Due to such homogeneity, optical microscopy failed to observe distinctive microstructural features at varying magnifications. Pores and surface contaminants are observable across the morphologies of all alloys, including carbon deposits.

**Figure 2:**
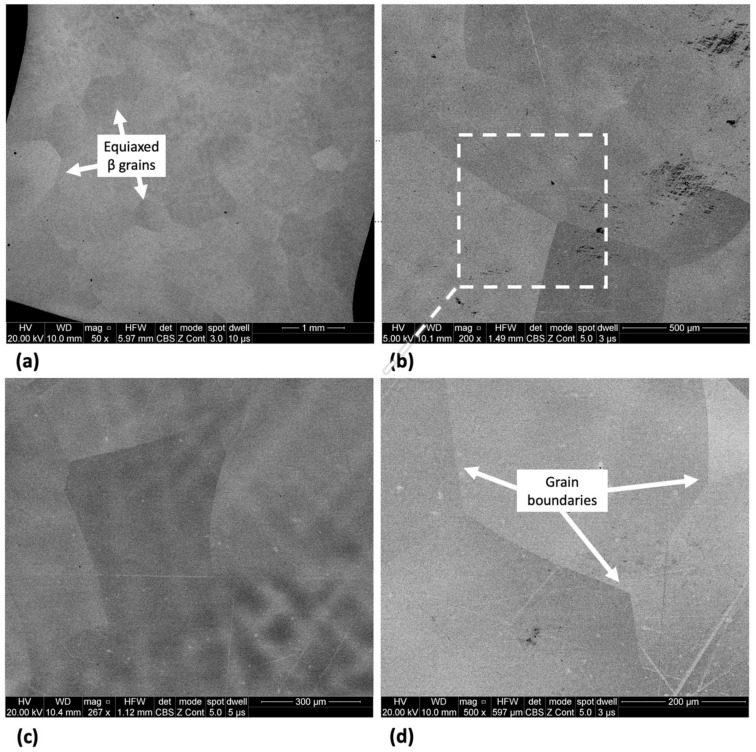
SEM micrographs of Ti-33Nb-3Ga alloy at various magnifications of (**a**) 50×, (**b**) 200×, (**c**) 267×, and (**d**) 500× showing distinct β-phase grains.

**Figure 3:**
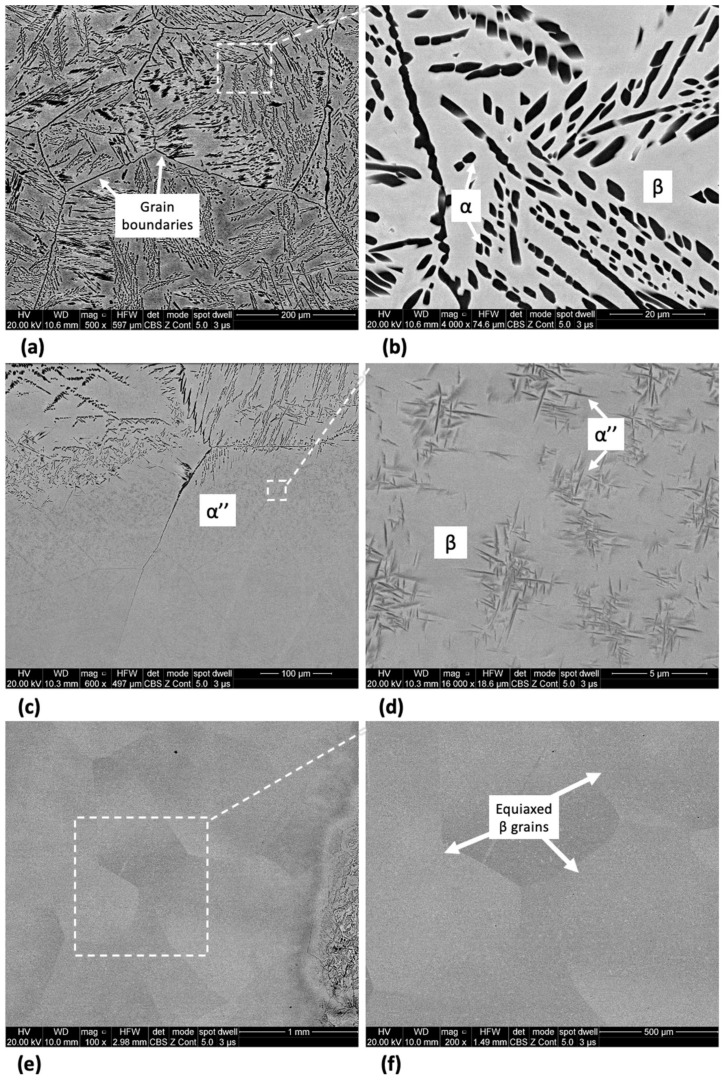
SEM micrographs of Ti-33Nb-5Ga alloy at various magnifications showing dendritic α-phase colonies (**a**,**b**), transformed martensitic phase (**c**,**d**), and equiaxed β-phase grains (**e**,**f**).

**Figure 4:**
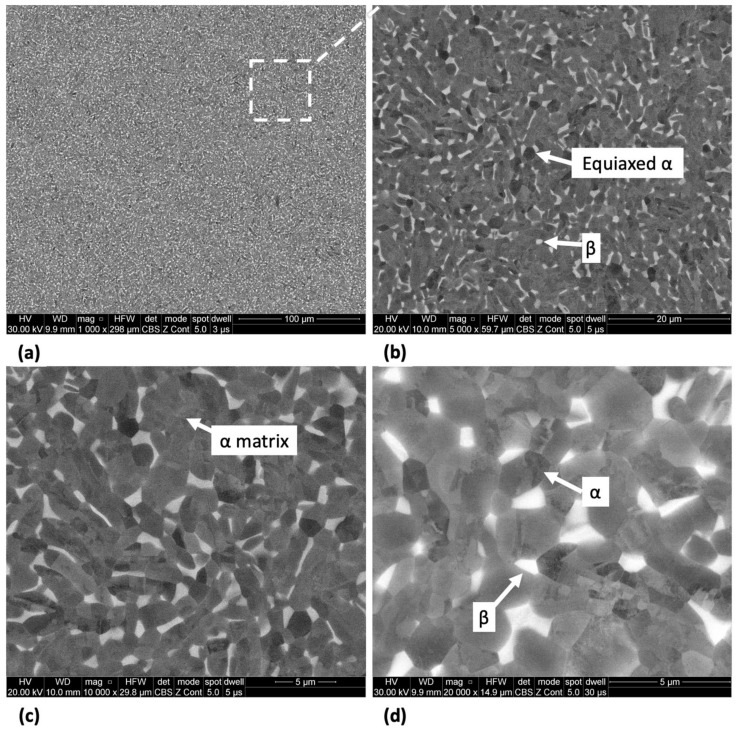
SEM micrographs of the Ti-6Al-4V alloy at magnifications of (**a**) 1000×, (**b**) 5000×, (**c**) 10,000×, and (**d**) 20,000× showing alpha equiaxed grains with minor β-phase grains.

The microstructure of Ti-33Nb-3Ga and Ti-33Nb-5Ga, together with the reference Ti-6Al-4V alloy, observed by SEM imaging is depicted in Figure 2, Figure 3, and Figure 4, respectively. SEM backscattered micrographs for the Ti-33Nb-3Ga alloy, shown in Figure 2a–d, depict a single-phase character consisting of equiaxed β-grains with grain sizes in the range of approximately 400 to 900 μm. Figure 3a–f illustrates the SEM backscattered micrographs for the Ti-33Nb-5Ga alloy, which consists of various phases dominated by dendritic and inter-dendritic regions (Figure 3a,b). High contrast, dendritic regions are evidence of α-phase colonies ranging from 3 to 6 μm, while lighter regions correspond to a β-phase equiaxed matrix. Grain boundaries are enriched in α-phase character for Ti-33Nb-5Ga, shown in Figure 3a,c. Martensitic transformation of the β-phase into α″-phase is also observed (Figure 3c,d). Moreover, single-phase β-type character is depicted in Figure 3e,f, which is comparable to the microstructure observed for the 3 wt% Ga sample. Grain sizes in the 5 wt% Ga alloy appear to diminish compared to the 3 wt% Ga alloy, with an average grain size of 500 μm. SEM images of the reference alloy depict uniform phase distribution with mostly homogenous grain sizes ranging from 1 to 4 μm, as observed in Figure 4a–d. The Ti-6Al-4V microstructure contains primary alpha equiaxed grains with transformed beta grains uniformly distributed in the alpha matrix. Shown in Figure 4d, the presence of transformed beta grains consisting of elongated alpha needles is additionally observed.

**Figure 5:**
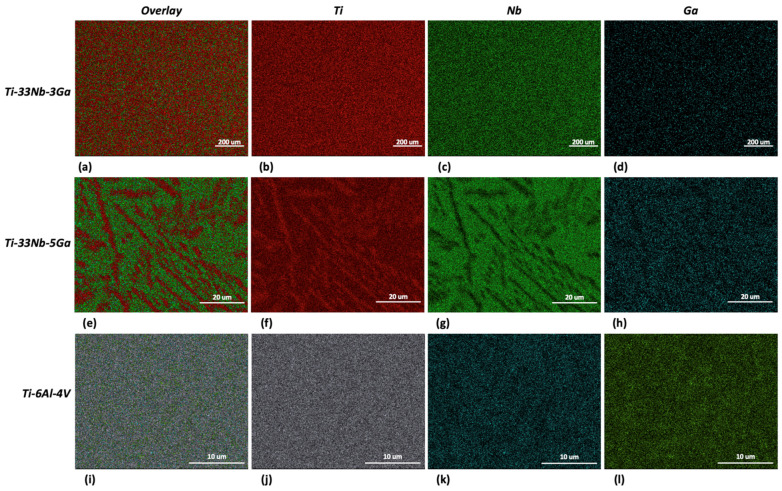
EDS mapping for: (**a**–**d**) Ti-33Nb-3Ga, (**e**–**h**) Ti-33Nb-5Ga, and (**i**–**l**) Ti-6Al-4V.

**Figure 6:**
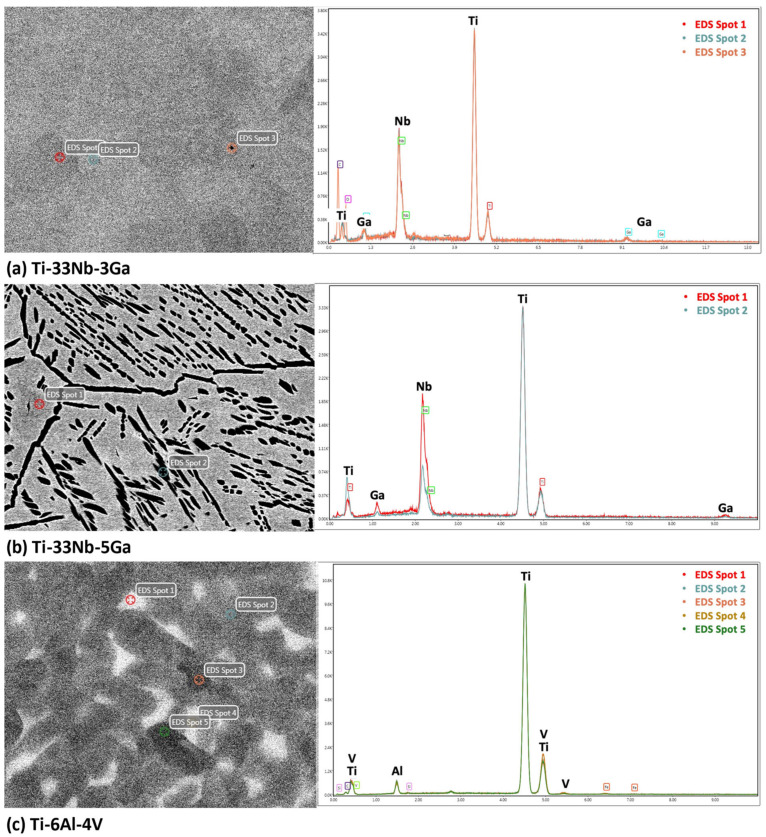
EDS spectrum analysis of the alloy samples indicating chemical composition of microstructural features and the overall alloy for: (**a**) Ti-33Nb-3Ga, (**b**) Ti-33Nb-5Ga, and (**c**) Ti-6Al-4V.

The results from the EDS analysis aid in elucidating the chemical composition and homogeneity of the developed alloys, as shown in Figure 5 and Figure 6. Elemental mappings of the Ti-33Nb-3Ga alloy reveal the presence of a chemically homogenous microstructure, depicted in Figure 5a–d. Such uniformity is also corroborated by the results in Figure 6a, where lighter and darker grains correspond to the same elemental composition. The 5 wt% Ga alloy demonstrates elementally enriched areas within the microstructure, shown by the EDS mapping in Figure 5e–h. Corroborating with this result, Figure 6b reveals that the darker, α-phase regions are enriched with titanium, while the β-phase is more elementally concentrated in both niobium and gallium. Furthermore, the Ti-6Al-4V reference alloy depicted a chemically homogenous microstructure from the elemental mappings in Figure 5i–l. The chemical composition analysis in Figure 6c further discloses the micrometric homogenous distribution of alloying elements among grains within this alloy. Revealed by EDS, the chemical composition reported for the 3 wt% alloy was 67.73 wt% Ti, 27.48 wt% Nb, and 4.80 wt% Ga, while the chemical composition of the 5 wt% alloy was indicated as 70.16 wt% Ti, 27.44 wt% Nb, and 2.40 wt% Ga. However, it is widely acknowledged that this method does not yield precise chemical composition analysis, where the results should be considered as indicative rather than definitive.

**Figure 7:**
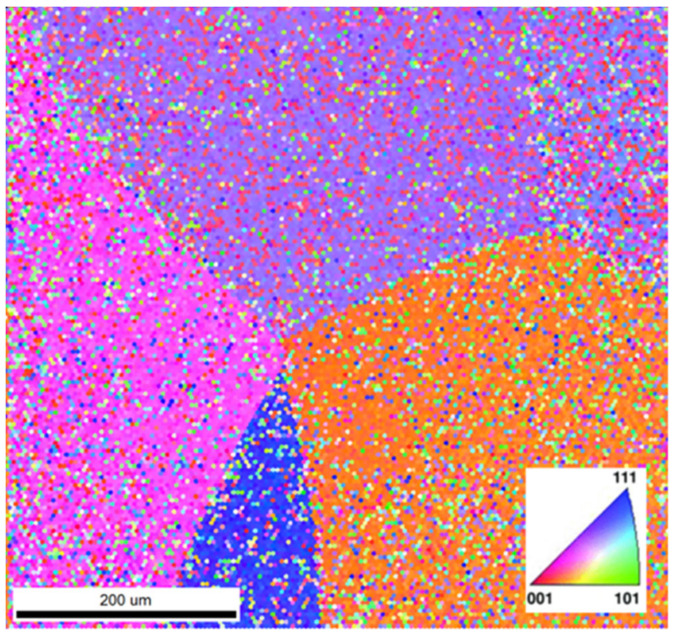
Inverse pole figure (IPF) map of the grains in Ti-33Nb-3Ga. Colours correspond to the Miller indices and therefore crystal structure orientations.

Electron backscatter diffraction (EBSD) and inverse pole figure (IPF) mapping of the β-phase grain structure, as exhibited for both the Ga-containing alloys, reveal the various orientations of the β phase crystal lattice, as shown in the IPF mapping of Ti-33Nb-3Ga in Figure 7. Congregations of colour are indicative of the same lattice planes in crystal lattices, where the Miller indices of the pink, purple, orange, and blue assemblies correspond to 011, 101, 110, and 102 planes in the cubic crystal, respectively. This indicates that the β-phase grains do not differ elementally, also confirmed by EDS in Figure 6a, and instead vary by the differing orientations of their crystal structure.

### 3.2. Mechanical Characterisation

#### 3.2.1. Microhardness

**Figure 8:**
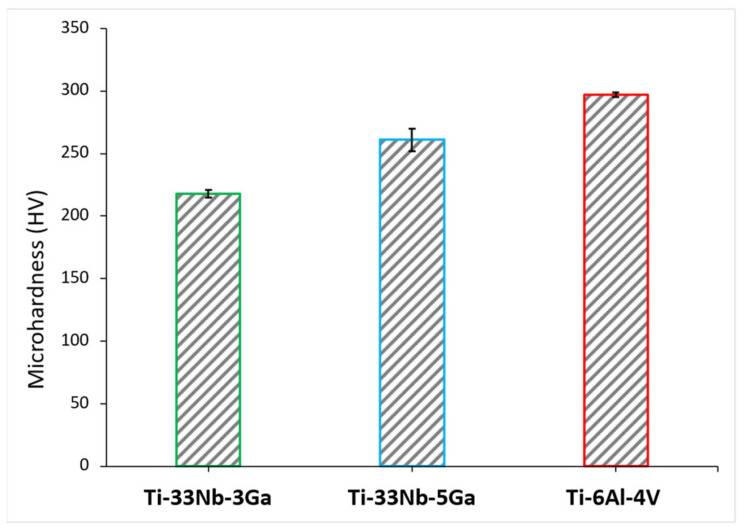
The mean Vickers microhardness (HV) for the developed alloys and the reference alloy (Ti-6Al-4V).

The variation in Vickers microhardness as a function of increasing gallium content (wt%) compared to the reference material is depicted in Figure 8, with values obtained for Ti-33Nb-3Ga, Ti-33Nb-5Ga, and Ti-6Al-4V being 218.0 ± 2.9, 260.9 ± 9.2, and 297.0 ± 1.9 HV, respectively. Increasing elemental concentrations of gallium from 3 to 5 wt% resulted in an approximate 20% increase in microhardness, indicating a possible strengthening effect from gallium addition. The microhardness result for Ti-6Al-4V is in accordance with values reported in the literature [30,31].

#### 3.2.2. Nanoindentation

**Figure 9:**
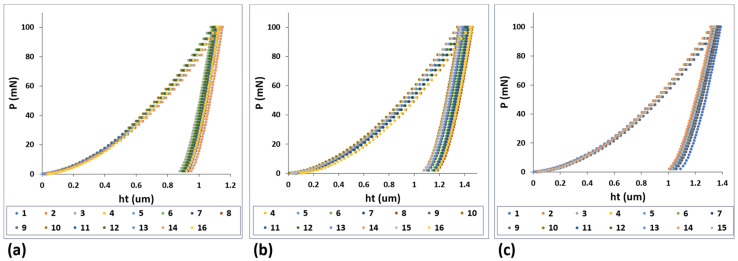
Load-displacement curves from nanoindentation results for: (**a**) Ti-33Nb-3Ga, (**b**) Ti-33Nb-5Ga, and (**c**) Ti-6Al-4V.

The typical load versus displacement curves as obtained from nanoindentation tests are illustrated in Figure 9 for the Ti-33Nb-3Ga, Ti-33Nb-5Ga, and Ti-6Al-4V alloys under a maximum load of 100 mN. The load-depth curves reveal the loading, holding, and unloading stages, where the elastic recovery process is observed in the latter.

**Figure 10:**
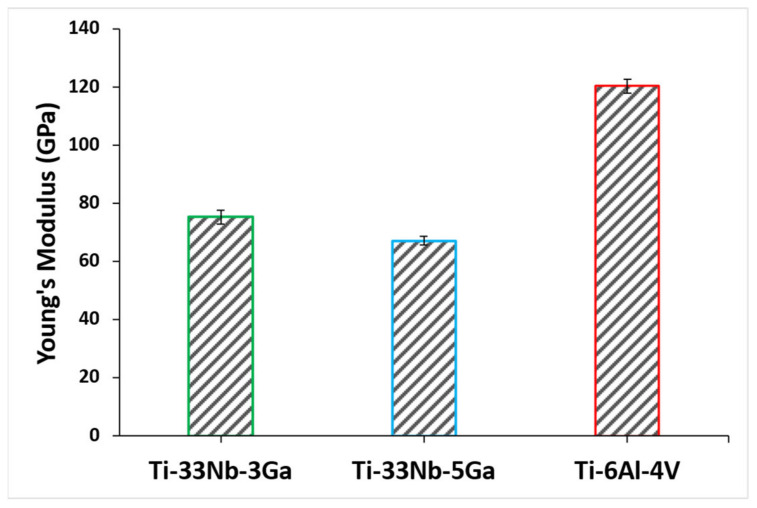
Young’s modulus of the developed alloys and reference material determined using nanoindentation testing.

The Young’s modulus results for the developed Ga-containing alloys together with the reference alloy are depicted in Figure 10, where the mean results and standard deviation are also reported in Table 3. The Young’s modulus of Ti-33Nb-3Ga and Ti-33Nb-5Ga were found as 75.4 ± 2.4 GPa and 67.2 ± 1.6 GPa, respectively. The measured Young’s modulus result for Ti-6Al-4V was 120.5 ± 2.3 GPa, which is in close agreement with reported values in the literature [32,33]. The addition of higher gallium concentrations from 3 to 5 wt% to the Ti-33Nb matrix led to a favourable decrease in the Young’s modulus by 11%. Notably, both of the developed alloys were found to have a significantly reduced Young’s modulus compared to the reference alloy, where the Young’s modulus for the 3 wt% Ga sample was 37% less than Ti-6Al-4V, and the 5 wt% Ga alloy was lowered as much as 44% compared to the reference alloy.

**Figure 11:**
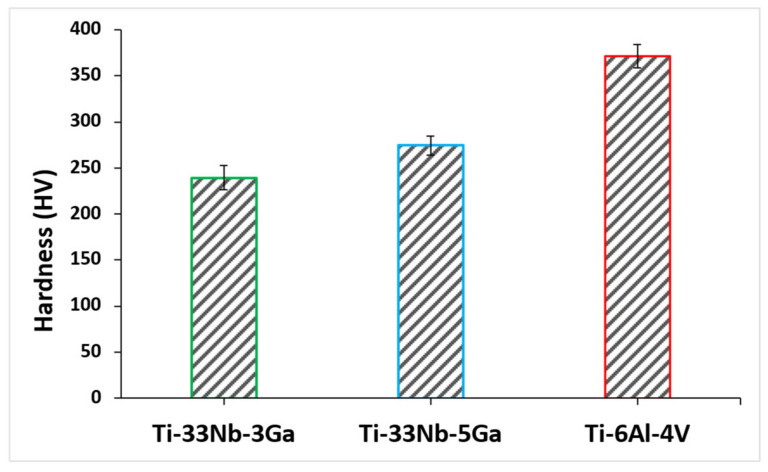
Nanohardness of the developed alloys and reference alloy obtained via nanoindentation testing.

The effect of gallium addition on the nanohardness for the respective alloy samples is shown in Figure 11. Nanohardness values exhibit a strengthening effect with increasing gallium content, where the measured values for Ti-33Nb-3Ga and Ti-33Nb-5Ga were 239.5 ± 13.6 HV and 274.3 ± 9.9 HV, respectively (Table 3). This result represents an increase in hardness of 14% between alloys. Comparatively, the reference alloy exhibited a nanohardness of 371.4 ± 13.0 HV, which is similarly reported throughout literature [34].

### 3.3. Biological Characterisation

#### 3.3.1. Antimicrobial Activity Assay

*S. aureus* and *P. aeruginosa* were selected for bacterial testing due to their clinical significance in implant-related infections [35]. Gram-positive *S. aureus* is a common cause of surgical site infections and is known for its ability to form biofilms on implant surfaces [36]. Gram-negative *P. aeruginosa* is notorious for its antibiotic resistance and role in chronic infections [37]. By choosing these two bacterial strains, evaluation of the antibacterial properties of the alloys against a broad spectrum of clinically significant pathogens is targeted, thereby demonstrating the potential applicability of the alloys in real-world medical settings.

**Figure 12:**
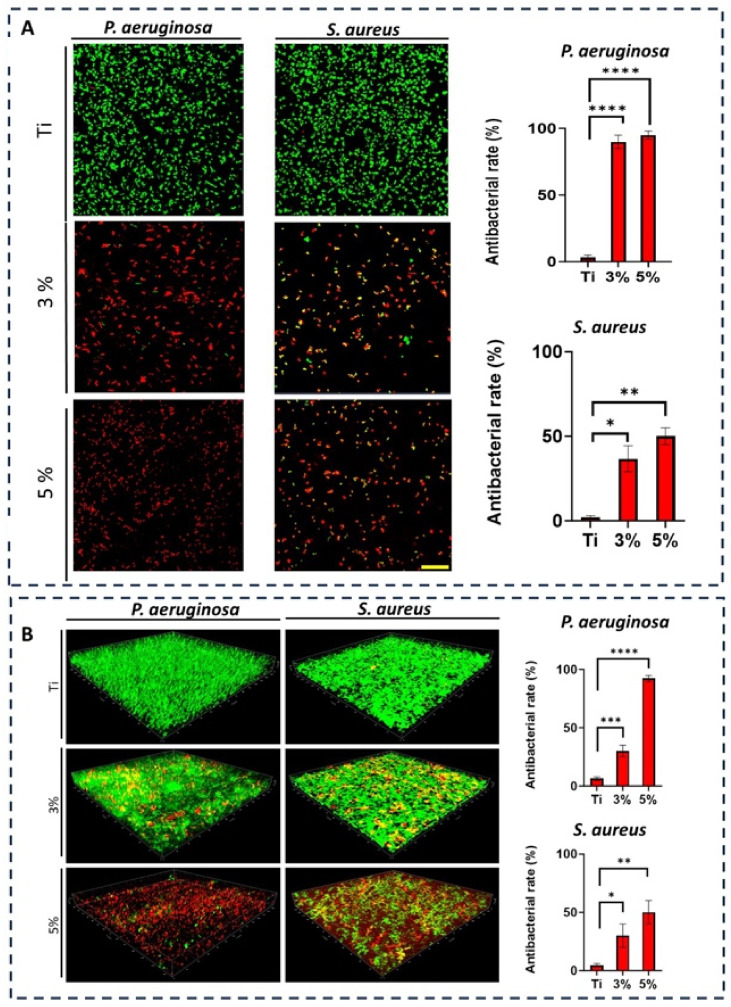
Ti-6Al-4V (labelled Ti), Ti-33Nb-3Ga (labelled 3%), and Ti-33Nb-5Ga (labelled 5%) antibacterial activities against Gram-negative *Pseudomonas aeruginosa* (*P. aeruginosa*) and Gram-positive *Staphylococcus aureus* (*S. aureus*). CLSM pictures and associated plots assessing the antibacterial efficiency of Ti, 3%, and 5% against *P. aeruginosa* and *S. aureus* for 6 h (**A**) and 24 h (**B**). Dead bacteria are red, while living bacteria are green. The scale bar measures 20 µm. *n* = 3 ± SD. * *p* < 0.1, ** *p* < 0.01, *** *p* < 0.001, **** *p* < 0.0001.

The in-vitro antibacterial activity of Ti-33Nb-3Ga and Ti-33Nb-5Ga specimens, together with the reference Ti-6Al-4V, against Gram-negative *P. aeruginosa* and Gram-positive *S. aureus* bacteria is reported in Figure 12. The antibacterial rate was quantified over 6 and 24 h, where both *P. aeruginosa* and *S. aureus* bacteria exhibited significant inhibition when directly seeded to the gallium-containing alloys. For Ti-33Nb-3Ga and Ti-33Nb-5Ga, the antimicrobial rate observed against *P. aeruginosa* over 6 h was 90 ± 5% and 95 ± 3%, respectively (Figure 12A), while Ti-6Al-4V demonstrated a very small rate of 3 ± 2%. Consequently, the inhibition of *S. aureus* bacteria over a 6 h period was measured at 36.7 ± 7.6% and 50 ± 5% for 3 wt% and 5 wt% gallium samples, respectively. Here, the bacterial inhibition exhibited by Ti-6Al-4V was merely 2 ± 1%. Furthermore, after 24 h, the antibacterial rate over *P. aeruginosa* bacteria was measured at 30 ± 1% for Ti-33Nb-3Ga, 92.3 ± 2.5% for Ti-33Nb-5Ga, and 6.3 ± 1.5% for Ti-6Al-4V, as shown in Figure 12B. For *S. aureus*, the bactericidal effect over a 24 h incubation indicates 30 ± 10% and 50 ± 10% inhibition by 3 and 5 wt% Ga-alloys, respectively, while Ti-6Al-4V exhibited a rate of 4.7 ± 1.5%.

The CLSM images of bacterial strains on the surfaces of the alloys were captured to further elucidate the antibacterial efficacies of the samples. Dead (red) and live (green) bacteria were observed using fluorescence staining. Figure 12A depicts the bacterial viability over 6-h incubation, while Figure 12B shows the Z-stack surface rendered 3D models depicting the bacterial biovolume density for *P. aeruginosa* and *S. aureus* at 24 h. Viability staining reveals a significantly high proportion of dead and metabolically inactive cells for both Ti-33Nb-3Ga and Ti-33Nb-5Ga alloys, where the 5 wt% sample exhibits abundant cell death. Notably, the reference Ti-6Al-4V alloy is predominated by live cells, indicating bacterial cell strains remained healthy. The results indicate that the Ti-33Nb-5Ga alloy achieved the highest percentage of nonviable cells in both Gram-negative and Gram-positive bacterial cells.

#### 3.3.2. Cytotoxicity Assay

**Figure 13:**
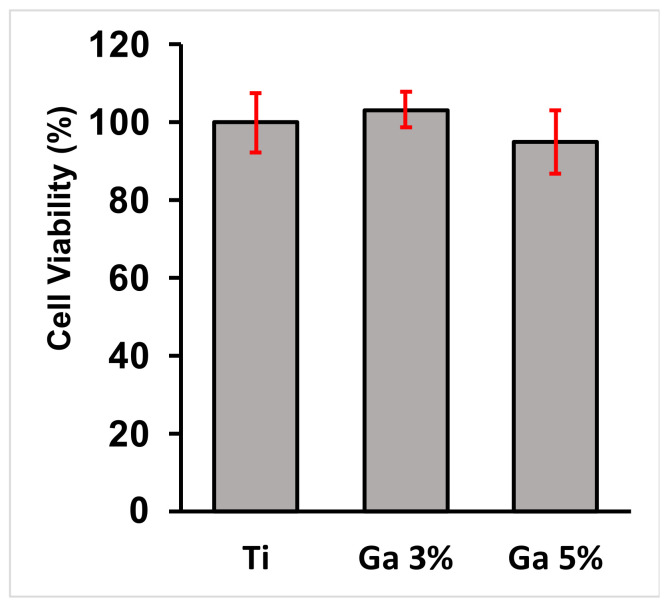
Cytocompatibility analysis of the alloys; values are representative of means and standard deviations. The assay showed that no toxic elements were released from the surface of the specimens as there was no significant difference (*p* < 0.05) between the designed alloys and the control alloy after 24 h.

The cell viability of the hGF cells cultivated onto the surfaces of the Ti-33Nb-3Ga and Ti-33Nb-5Ga alloys, together with the control Ti-6Al-4V, are depicted in Figure 13 and in Table 4. The cell viability for the control group (Ti-6Al-4V) was normalised and compared to that of the 3 wt% and 5 wt% Ga samples, reported at 103.1 ± 4.6% and 94.9 ± 8.2%, respectively. Among the tested alloys, there is no significant difference when comparing the cell viability obtained by the Ti-33Nb-3Ga and Ti-33Nb-5Ga alloy specimens to the control surface (Ti-6Al-4V) (*p* < 0.05). Therefore, high cell proliferation and no cytotoxicity was observed on hGF cells in direct contact with the specimens over 24 h.

**Figure 14:**
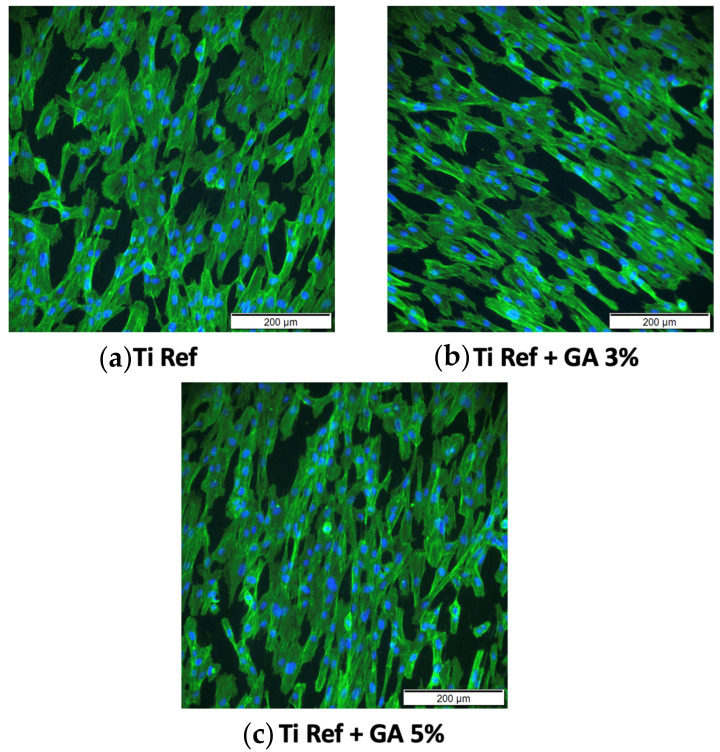
Cytoskeleton organisation and cell morphology imaged with an inverted microscope at 200 μm. Images depict stained F-actin (green) and nucleus (blue) for: (**a**) Ti-6Al-4V control, (**b**) Ti-33Nb-3Ga, and (**c**) Ti-33Nb-5Ga alloys.

Fluorescence images of the cell morphology and cytoskeleton distribution on the surface of the titanium samples are depicted in Figure 14. Images depict staining of actin cytoskeleton and nucleus with FITC conjugated phalloidin (green) and DAPI (blue), respectively. Cells directly exposed to alloys depict a healthy, typical morphology with a prominent nucleus, elongated fibroblastic spindle-shaped morphology, and F-actin filamentous stress fibres in a well-organised and defined structure of parallel bundles along the cellular axis. No significant difference was observable between Ti-33Nb-xGa specimens to the cytoskeleton distribution and cell morphology on the surface of the Ti-6Al-4V control. In addition, the cytoskeleton organisation is not affected by increasing elemental additions of gallium to the master alloy, evidenced by an absence of changed cell morphology and the lack of filament condensing. These results corroborate with the assay results in Figure 13 and Table 4. Images of stained cells in Figure 14 therefore clearly suggest the presence of healthy cells for the F-actin cytoskeleton and nucleus, indicating that the surfaces of the alloys are not cytotoxic.

## 4. Discussion

### 4.1. Microstructural and Mechanical Evaluation

Minor additions of gallium (3 and 5 wt%) to new-generation β-phase Ti-Nb alloys were investigated in this work to address the alloys’ inability to prevent biofilm formation while also maintaining low stiffness values. Microstructural evaluation was conducted to reveal the expected mechanical performance and suitability of the alloys as biomaterials. The microstructure of the Ti-33Nb-3Ga alloy depicted equiaxed β-phase grains, with full retainment of a single BCC β-phase despite the addition of gallium. Retainment of a single β-phase is expected, as beta stabilisation is obtained from alloying with Nb, in addition to slow cooling rates maintained during casting. Consequently, the 5 wt% Ga alloy depicted slight retainment of BCC β-phase in the form of equiaxed grains shown in Figure 3e,f; however, the microstructure of this alloy was dominated by dendritic and interdendritic regions of α-phase colonies precipitated in the β-phase matrix. As this alloy contains greater concentrations of elemental gallium, this was likely an anticipated result due to the α-phase stabilisation provided by Ga. The size of dendrites directly correlates to the cooling rate of the alloy, where sufficiently fast cooling rates lead to martensitic transformation, as shown in Figure 3c,d. As observed for Ti-33Nb-3Ga and parts of Ti-33Nb-5Ga, a retained single β-phase was detected, and is in agreement with Alberta et al. [8] for all alloys of Ti-45Nb-xGa (x = 2, 4, 6, 8 wt%). Though there is no evidence of alpha phases or martensitic transformation in their work, other new generation Ti-Nb alloys have shown dendritic features and α-phase transformation [38,39,40]. The microstructure of the reference alloy (Ti-6Al-4V), illustrated in Figure 4a–d, is also observed throughout literature, evidencing the α-β character [41,42].

The intricate correlation between microstructure and mechanical properties aids in informing the underlying mechanisms that govern the material properties of the developed alloys. Optimised Young’s modulus values are known to be obtained by retaining a β-phase microstructure, where gallium is an α stabiliser that may disrupt the beta phase stability by influencing titanium phase transformation (${Ti}_{\alpha \to \beta }$ = 882 °C). In contrast to this notion, the 5 wt% Ga alloy with multiple phases depicted the best reduction in Young’s modulus, with a stiffness of 67.2 ± 1.6 GPa. This indicates that titanium alloys with multiple phases can decrease the Young’s modulus, as also reported by [43]. The occurrence of martensitic transformation into the α″-phase has previously been recognized for its contribution to reducing the Young’s modulus, and, when coupled with the presence of equiaxed grains in the β-phase, is believed to underpin this observed outcome [16,44]. Furthermore, the replacement of titanium and niobium elements by smaller gallium atoms brings atoms closer together in the beta crystal lattice, potentially instigating further changes to the Young’s modulus. Moreover, the improvements to the Young’s modulus from Ti-33Nb-3Ga and Ti-33Nb-5Ga by 37% and 44%, respectively, over the reference material is expected to significantly alleviate stress-shielding effects and bone resorption, resulting in minimised revision surgeries and diminished implant failure compared to Ti-6Al-4V. The microstructure of the bimodal Ti-6Al-4V alloy is abundant in primary α-phase grains with some retained transformed beta phases, which contributes to its high Young’s modulus (Figure 4). Compared to earlier investigations on β-type Ti-Nb alloys, including [8,24,25,45,46,47,48], the current outcomes improve on stiffnesses results previously reported. Alberta et al. [8] tested a series of Ti-45Nb-xGa (x = 2, 4, 6, 8 wt%) alloys and observed Young’s modulus values within the range of 73.0 to 82.5 GPa.

The presence of light and dark β-phase grains does not correlate with elemental differences, as previously noted in the case of α-phase dendrites (richer in Ti) surrounded in β-phase matrix (richer in Nb and Ga) for Ti-33Nb-5Ga (Figure 5 and Figure 6). The IPF map depicted in Figure 7 indicates that variations in grains correspond to differences in the crystal structure orientation. Notably, the bonding force between atoms is restricted to the crystal structure, in addition to the distance between atoms, and is known to affect the Young’s modulus. As the distance between atoms in Ti-33Nb-3Ga and Ti-33Nb-5Ga alloys have not been affected by heat treatment processes or plastic deformation, the Young’s modulus values are believed to have been influenced by the crystal lattice orientations within the alloys. These orientations, and therefore their effect on the Young’s modulus, are postulated to have been induced by Ga alloying additions on the distances between atoms.

The microhardness and nanohardness values of the novel alloys closely approach the hardness level of the reference alloy, exhibiting a notable consistency with results documented in the literature for Ti-Nb alloys characterized by a predominant β-phase matrix [8,25]. Furthermore, results show improved hardness values over numerous previously reported Ti-Nb alloys, including [2,49,50,51], where a clear strengthening effect is exhibited when alloying additions of gallium to the matrix of the Ti-33Nb master alloy is increased. Grain refinement is observed for the β-phase character of the Ti-33Nb-3Ga and Ti-33Nb-5Ga microstructures, where grain refinement has previously been reported in literature to influence the hardness properties of alloys. Shown in Figure 2 and Figure 3e,f, both alloys possess equiaxed β-grains, where the 5 wt% Ga alloy contains finer average grain sizes. The microhardness results for Ti-33Nb-3Ga and Ti-33Nb-5Ga from both Vickers microhardness (218 ± 2.9 HV and 260.9 ± 9.2 HV, respectively) and nanoindentation (239.5 ± 13.6 HV and 274.3 ± 9.9 HV, respectively) methods are in agreement that Ti-33Nb-3Ga possesses a lower hardness than that of the Ti-33Nb-5Ga alloy. Hardness results therefore reveal a strengthening effect of gallium addition to the Ti-33Nb master alloy. This behaviour can be correlated to the reduced size of the β-phase equiaxed grains in this alloy, where it is believed that the presence of Ga atoms reduces the surface energy mismatch among crystal structure surfaces. This pinning effect halters the growth of grains, lessening their size and subsequently increasing the homogeneity of the microstructure. Furthermore, the presence of precipitated dendritic α-phases in Ti-33Nb-5Ga (Figure 3a–c), in addition to martensitic transformation, also contributes to an increase in hardness compared to the single β-phase exhibited in the 3 wt% Ga alloy. An augmented concentration of gallium is believed to have led to a higher occurrence of alpha precipitations, subsequently leading to an increase in hardness. It is probable that the α-phase played a vital role in strengthening the 5 wt% Ga alloy by impeding dislocations. Such hardness findings serve as vital indicators of the aptness of these alloys for biomedical implant applications. When combined with the Young’s modulus results, both alloys show promising mechanical properties, with particular emphasis on the 5 wt% variant, showcasing mechanical properties that either surpass or, in the case of hardness, are just short of being at parity with the currently employed clinical Ti-6Al-4V alloy. However, analysis of the wear and tribocorrosion properties is also necessitated to evaluate and further inform the life span of the alloys when implanted, and their subsequent suitability [12].

### 4.2. Biological Evaluation

The emergence of new generation β-phase Ti-Nb alloys that show excellent mechanical properties and biocompatibility is limited by their inability to inhibit bacterial infections, which may ultimately result in implant failure. The significant antibacterial properties of both Ti-33Nb-3Ga and Ti-33Nb-5Ga alloys against Gram-negative *P. aeruginosa* and Gram-positive *S. aureus* bacteria are revealed in Figure 12. Both alloys depict significant antimicrobial action, with the best results against *P. aeruginosa* cells over 6 h. Antibacterial activity up to 90 ± 5% for the 3 wt% alloy was observed, in addition to 95 ± 3% for the 5 wt% alloy. At 24 h incubation, the potency of Ti-33Nb-3Ga against *P. aeruginosa* decreased to 30 ± 5%, while the Ti-33Nb-5Ga alloy remained unchanged at 92.3 ± 2.5%. This indicates that the 5 wt% Ga alloy exhibits a significantly elevated and prolonged antimicrobial potency against Gram-negative bacteria, regardless of time. This includes as much as a 208% increase in antimicrobial properties over that of Ti-33Nb-3Ga. Furthermore, inhibition results against *S. aureus* depict the same trends, confirming that increased alloying additions of gallium are advantageous to the antimicrobial properties. Notably, the smaller antimicrobial impact observed for all alloys in *S. aureus* bacteria may be attributed to the more complex cell wall in Gram-negative bacteria [52]. This may slow gallium ion penetration and limit efflux mechanisms, ultimately leading to a smaller concentration of gallium in the intracellular region, and therefore smaller inhibitions.

Both Ga-containing alloys exhibit a significant improvement in the antibacterial potency when compared to the presently employed Ti-6Al-4V implant material. This includes surpassing the antimicrobial rate of Ti-6Al-4V by a substantial margin. The mechanism underlying the observed antibacterial effect in Ga-containing alloys is hypothesized to involve a “Trojan horse” strategy of bacterial inhibition, which has been extensively studied [53,54,55,56]. Gallium ions (Ga^3+^) contain an analogous electron affinity, ionisation potential, and ionic radius with ferric ions (Fe^3+^), potentially leading them to be mistaken for Fe^3+^ and binding strongly to iron-binding proteins crucial in bacterial metabolic and signalling processes [17,50,53]. Bacterial cells produce siderophores to uptake iron, and Ga^3+^ competes with Fe^3+^ for binding to siderophores and vital proteins and enzymes [53]. Gallium is not redox-active; when bound to iron-binding proteins, it can disrupt various iron-dependent redox pathways, thereby compromising bacterial cell function and survival [53,54,56]. By comparing their antibacterial efficacy, it becomes evident that Ti-33Nb-3Ga and Ti-33Nb-5Ga possess a superior capacity to inhibit biofilm formation, consequently reducing the likelihood of implant failure and the need for revision surgeries in comparison to the currently utilized Ti-6Al-4V.

Although there is a lack of literature existing for gallium-based materials, with a particular gap relating to gallium-based alloy systems, the antibacterial properties of Ti-33Nb-3Ga and Ti-33Nb-5Ga represent improved values compared to those reported previously for gallium material systems in literature, including [13,21,52,57,58,59,60]. Notably, most of the literature examines materials with gallium coatings and are therefore expected to be more potent over shorter times compared to metallurgically added Ga. However, the results in this study show stronger inhibition of bacteria over previous gallium-coated systems. For example, commercial titanium alloys coated with both Ga(NO_3_)_3_ and chelating agent l-Cysteine (GaCis) in addition to Ga(NO_3_)_3_ coated with chelating agent oxalic acid (GaOss) depicted a 27–35% decrease in *A. baumannii* [21,57]. The results in this research show an improvement in this value by as much as 242%. This is particularly notable, as alloys with metallurgical additions of gallium are more likely to retain their antimicrobial efficacies, while gallium-coated biomaterials are generally more susceptible to wear in the body, consequently diminishing their potency over time. Furthermore, to the author’s knowledge, Cochis et al. [13] is the only study to have examined the antibacterial activity of titanium alloys with elemental gallium additions. In this work, 1, 2, and 24 wt% Ga additions to Ti-Al-Zr-Si observed a reduction in viability at a maximum of 80–95%. However, the master alloy would likely exhibit high stiffness values due to the absence of niobium, where the alloy also contains harmful alloying additions of Al. The developed Ti-33Nb-5Ga alloy therefore demonstrates antimicrobial results that are not only comparable but superior to that reported in [13].

The investigation of in vitro biocompatibility and cell proliferation yielded favourable results, indicating the absence of cytotoxicity in all alloys. The MTT assay was used to assess cell viability and proliferation. It is based on the conversion of the yellow tetrazolium salt (MTT) to purple formazan crystals by metabolically active cells [61]. The viability of the hGF cell line in direct contact with alloy surfaces over 24 h was evaluated. Results in Figure 13 reveal that both Ti-33Nb-3Ga and Ti-33Nb-5Ga support cell viability that does not significantly differ from the control Ti-6Al-4V (*p* < 0.05), with mean cell proliferations of 103.1 ± 4.6% and 94.9 ± 8.9%, respectively. These conclusions are afforded as the statistical analysis of the p-value for both alloys are less than 0.05. Furthermore, cell morphology and cytoskeleton distribution on the surfaces of the alloys was imaged after F-actin staining procedures, as shown in Figure 14. The F-actin cytoskeleton is a critical cell structure to cellular functions such as division, intracellular transport, signalling pathways, and gene expression, as well as in mechano-transduction and motility mechanisms associated with differentiation [62]. Moreover, F-actin is also used as a marker for cell adhesion [63]. Due to the stability afforded by F-actin and its active collaboration with cellular function, as well as its responsiveness to surrounding environment, the F-actin cytoskeleton is a preliminary indicator of cytotoxicity [62]. Because no deleterious effects to the structure were evidenced in cell staining, such as changes to cell morphology and condensing, alloys depict obvious biocompatibilities with no cytotoxicity. Both alloys show images consistent with healthy cell morphology and cytoskeleton, such as a prominent nucleus and elongated fibroblastic spindle-shaped morphology. Biocompatibility is a fundamental requirement for the clinical application of biomaterials in orthopaedics. It refers to the ability of a biomaterial to perform its intended function without causing toxic or harmful effects on biological systems, while also eliciting an appropriate host response in the specific context of its use [64]. The results from this study indicate that the alloy surfaces have no cytotoxic effect on the growth of hGF cells, therefore informing the biocompatibility of the alloys for potential implantation in orthopaedic biomedical applications. These findings are in accordance with previous studies, where gallium has previously been reported to be a safe metal for use in biomedical applications by [65,66,67,68]. For example, metallurgical addition of gallium in 1–2 wt% up to 23 wt% observed no cytotoxicity in human fetal progenitor and human osteosarcoma cell lines [13]. Further studies may be necessary to comprehensively assess the suitability of these materials for clinical use. Long-term in vivo studies and clinical trials are essential to confirm these preliminary findings and ensure safety and efficacy in orthopaedics applications.

The inherent limitations exhibited throughout this study, as manifested in the results, provide valuable insights for their prospective resolution in future research endeavours. Due to the investigation of merely two novel alloys, trends correlated to increasing Ga content could not be entirely elucidated. Further exploration of various gallium compositions added to Ti-33Nb is therefore warranted with a focus on comparing the outcomes to the master alloy devoid of any gallium. In addition, size restraints of the cast alloys prevented tensile testing to evaluate a greater range of mechanical properties, such as yield strength, ultimate tensile strength, ductility, and elastic energy. These properties would better inform the suitability of the alloys as biomaterials, where collection of such data would benefit future studies. Furthermore, although the mechanical properties of both alloys were exceptionally encouraging, the effect of subjecting alloys to different processing parameters could show further improvements in mechanical properties. It is widely recognised that a reduction in Young’s modulus can be observed by controlling thermomechanical treatments and, therefore, the phase transformations. This could potentially result in further optimisation of the mechanical properties reported in this study. Finally, biocompatibility was assessed over 24 h and revealed that no cytotoxicity was exhibited for either alloy. However, further validation of this outcome could be investigated by measuring the effect of the alloys over a sustained time frame, and within different cell lines—for example, in human fetal progenitor and human osteosarcoma cell lines.

## 5. Conclusions

Two novel alloys with minor gallium addition (Ti-33Nb-3Ga and Ti-33Nb-5Ga) were developed and studied in terms of their microstructural, mechanical, antibacterial, and cytotoxic properties. The microstructures display homogenous equiaxed β-phase grains, whereby increasing Ga content to 5 wt% led to precipitation of dendritic α-phase colonies rich in titanium surrounded by a β-phase matrix enriched with niobium and gallium. Martensitic transformation to α″ was also observed in Ti-33Nb-5Ga. The homogenous, equiaxed BCC β-phase grains show differences in their crystal structure orientation, evidenced by IPF mapping. Furthermore, grain refinement was observed from increasing Ga content and is believed to have influenced the hardness of alloys. The developed Ga-containing alloys offer Young’s modulus (75.4 and 67.2 GPa) significantly lower than that of medical grade Ti-6Al-4V, as much as 44%. These results inform the suitability of both alloys, notably the 5 wt% Ga alloy, as implant biomaterials by possessing stiffness values closer to cortical bone, where stress shielding effects and therefore implant failure would be minimised. Moreover, the microhardness (218.0 and 260.9 HV) and nanohardness (239.5 and 274.3 HV) results reveal the strengthening effect of gallium addition to the Ti-33Nb master alloy, with results slightly lower than that of commercial Ti-6Al-4V. Furthermore, the presence of gallium, even in small amounts (3, 5 wt%), in the titanium master alloy produced highly efficient antibacterial function, with the best results being against *P. aeruginosa* cells over 6 h. Antibacterial activity up to 90 ± 5% for the 3 wt% alloy was observed, in addition to 95 ± 3% for the 5 wt% alloy. These results show a substantial improvement over the small antimicrobial rate of Ti-6Al-4V, which was only up to 6.3 ± 1.5%. After 24 h, the potency of Ti-33Nb-3Ga decreased, while the Ti-33Nb-5Ga alloy remained unchanged at 92.3 ± 2.5%. These results suggest that the 5 wt% Ga alloy exhibits a higher and more prolonged antimicrobial potency. From CLSM images, both alloys not only inhibit bacterial growth but also induce cell death in both Gram-negative and Gram-positive bacteria. Furthermore, each of the alloys also depict no cytotoxicity in the presence of hGF cell lines. This suggests that biofilm formation, revision surgeries, and implant failure would likely be minimised compared to conventional clinical Ti-6Al-4V. These results, paired with the mechanical properties and the exclusion of any toxic elements, indicate the potential application of both alloys as promising biomedical materials that could improve patient outcomes.

## Figures and Tables

**Table 1:** Chemical composition of Ti-33Nb-xGa (x = 3, 5 wt%) alloys in wt%, provided by American Elements.

Alloy	Elemental Composition (wt%)		
	Ti	Nb	Ga
Ti-33Nb-3Ga	63.9968	32.9835	2.9997
Ti-33Nb-5Ga	61.9969	32.9835	4.9995

**Table 2:** Chemical composition of Ti-6Al-4V alloy in wt%, provided by Dynamet.

Alloy	Elemental Composition (wt%)		
	Ti	Al	V
Ti-6Al-4V	89.8030	6.0000	4.0050

**Table 3:** Mean ± standard deviation of the Young’s modulus and nanohardness results for the developed alloys together with the reference material as determined by nanoindentation.

Alloy	Mean Young’s Modulus (GPa)	Mean Nanohardness (HV)
Ti-33Nb-3Ga	75.4 ± 2.4	239.5 ± 13.6
Ti-33Nb-5Ga	67.2 ± 1.6	274.3 ± 9.9
Ti-6Al-4V	120.5 ± 2.3	371.4 ± 13.0

**Table 4:** Cytotoxicity assay results, described as means and standard deviations. The *p*-value is reported, where no significant difference (*p* > 0.05) is observed between alloys.

	Ti-6Al-4V Ref (Normalised)	Ti-33Nb-3Ga	Ti-33Nb-5Ga
Mean Cell Proliferation	100	103.1	94.9
STDEV	7.6	4.6	8.2
*p*-value	-	0.414	0.29

## Data Availability

The original contributions presented in the study are included in the article, further inquiries can be directed to the corresponding author.
